# Neutralizing immunity against coronaviruses in Tanzanian health care workers

**DOI:** 10.1038/s41598-024-55989-4

**Published:** 2024-03-06

**Authors:** Godfrey Barabona, Isaac Ngare, Doreen Kamori, Lilian Nkinda, Yusuke Kosugi, Ambele Mawazo, Rayi Ekwabi, Gloria Kinasa, Harrison Chuwa, The Genotype to Phenotype Japan (G2P-Japan) Consortium, Keita Matsuno, Naganori Nao, Hirofumi Sawa, Shinya Tanaka, Masumi Tsuda, Lei Wang, Yoshikata Oda, Zannatul Ferdous, Kenji Shishido, Takasuke Fukuhara, Tomokazu Tamura, Rigel Suzuki, Saori Suzuki, Hayato Ito, Yu Kaku, Naoko Misawa, Arnon Plianchaisuk, Ziyi Guo, Alfredo A. Hinay, Keiya Uriu, Jarel Elgin M. Tolentino, Luo Chen, Lin Pan, Mai Suganami, Mika Chiba, Ryo Yoshimura, Kyoko Yasuda, Keiko Iida, Naomi Ohsumi, Adam P. Strange, Shiho Tanaka, Kazuhisa Yoshimura, Kenji Sadamasu, Mami Nagashima, Hiroyuki Asakura, Isao Yoshida, So Nakagawa, Kotaro Shirakawa, Akifumi Takaori-Kondo, Kayoko Nagata, Ryosuke Nomura, Yoshihito Horisawa, Yusuke Tashiro, Yugo Kawai, Kazuo Takayama, Rina Hashimoto, Sayaka Deguchi, Yukio Watanabe, Ayaka Sakamoto, Naoko Yasuhara, Takao Hashiguchi, Tateki Suzuki, Kanako Kimura, Jiei Sasaki, Yukari Nakajima, Hisano Yajima, Takashi Irie, Ryoko Kawabata, Kaori Tabata, Terumasa Ikeda, Hesham Nasser, Ryo Shimizu, M. S. T. Monira Begum, Michael Jonathan, Yuka Mugita, Otowa Takahashi, Kimiko Ichihara, Chihiro Motozono, Takamasa Ueno, Mako Toyoda, Akatsuki Saito, Maya Shofa, Yuki Shibatani, Tomoko Nishiuchi, Kei Sato, Bruno Sunguya, Takamasa Ueno

**Affiliations:** 1 Division of Infection and Immunity, Joint Research Center for Human Retrovirus Infection, Kumamoto University, Kumamoto, Japan; 2 Department of Microbiology and Immunology, Muhimbili University of Health and Allied Sciences, Dar es Salaam, Tanzania; 3 Collaboration Unit for Infection, Joint Research Center for Human Retrovirus Infection, Kumamoto University, Kumamoto, Japan; 4 Division of Systems Virology, Department of Microbiology and Immunology, The Institute of Medical Science, The University of Tokyo, Tokyo, Japan; 5 Graduate School of Medicine, The University of Tokyo, Tokyo, Japan; 6 Amana Regional Referral Hospital, Dar es Salaam, Tanzania; 7 Aga Khan Hospital, Dar es Salaam, Tanzania; 8 Graduate School of Frontier Sciences, The University of Tokyo, Kashiwa, Japan; 9 International Research Center for Infectious Diseases, The Institute of Medical Science, The University of Tokyo, Tokyo, Japan; 10 International Vaccine Design Center, The Institute of Medical Science, The University of Tokyo, Tokyo, Japan; 11 CREST, Japan Science and Technology Agency, Kawaguchi, Japan; 12 Department of Community Health, Muhimbili University of Health and Allied Sciences, Dar es Salaam, Tanzania; 13 Hokkaido University, Sapporo, Japan; 14 University of Tokyo, Tokyo, Japan; 15 Tokyo Metropolitan Institute of Public Health, Tokyo, Japan; 16 Tokai University, Tokyo, Japan; 17 Kyoto University, Kyoto, Japan; 18 Hiroshima University, Higashihiroshima, Japan; 19 Kyushu University, Fukuoka, Japan; 20 Kumamoto University, Kumamoto, Japan; 21 University of Miyazaki, Miyazaki, Japan

**Keywords:** Microbiology, Medical research

## Abstract

The ongoing vaccination efforts and exposure to endemic and emerging coronaviruses can shape the population's immunity against this group of viruses. In this study, we investigated neutralizing immunity against endemic and emerging coronaviruses in 200 Tanzanian frontline healthcare workers (HCWs). Despite low vaccination rates (19.5%), we found a high SARS-CoV-2 seroprevalence (94.0%), indicating high exposure in these HCWs. Next, we determined the neutralization capacity of antisera against human coronavirus NL63, and 229E, SARS-CoV-1, MERS-CoV and SARS-CoV-2 (including Omicron subvariants: BA.1, BQ.1.1 and XBB.1.5) using pseudovirus neutralization assay. We observed a broad range of neutralizing activity in HCWs, but no neutralization activity detected against MERS-CoV. We also observed a strong correlation between neutralizing antibody titers for SARS-CoV-2 and SARS-CoV-1, but not between other coronaviruses. Cross-neutralization titers against the newer Omicron subvariants, BQ.1.1 and XBB.1.5, was significantly reduced compared to BA.1 and BA.2 subvariants. On the other hand, the exposed vaccinated HCWs showed relatively higher median cross-neutralization titers against both the newer Omicron subvariants and SARS-CoV-1, but did not reach statistical significance. In summary, our findings suggest a broad range of neutralizing potency against coronaviruses in Tanzanian HCWs with detectable neutralizing immunity against SARS-CoV-1 resulting from SARS-CoV-2 exposure.

## Introduction

Humans are exposed to various human coronaviruses (HCoVs) and diverse zoonotic coronaviruses, which can potentially trigger adaptive immune responses cross-reacting with other coronaviruses, including the Severe Acute Respiratory Syndrome Coronavirus 2 (SARS-CoV-2)^1,2^. Data from human and animal studies indicated that immunity induced by SARS-CoV-2 infection and vaccination can cross react with other coronaviruses suggesting the trainability potential of coronaviruses heterologous immunity^3^. The recent SARS-CoV-2 pandemic and widespread vaccination efforts may further influence our adaptive immune responses against both endemic and emerging coronaviruses. Therefore, heterologous immunity against coronaviruses could play a role in favorably shaping our interactions with present and future coronaviruses.

In Africa, studies have shown substantial proportion of cross-reactive immunity against SARS-CoV-2 in pre-pandemic samples ^2,4,5^. This region has also documented the circulation of various coronaviruses among different bat species^6^. Moreover, domesticated camels, especially in Eastern and Northern Africa, exhibit a high prevalence of anti-Middle Eastern Respiratory Syndrome Coronavirus (MERS-CoV) IgG antibodies^7–9^. In the recent SARS-CoV-2 pandemic, official data suggest a relatively milder impact in terms of infection and mortality rates, especially in Sub-Saharan Africa^10^. However, some studies propose that SARS-CoV-2 infections were higher even early during the pandemic^11–13^, and its impact is potentially underestimated^14^. Nonetheless, coronavirus infections and the nature of immune responses in the African population remain largely understudied. Therefore, understanding emerging immunity against coronaviruses in this population is important to the development of strategies aimed at controlling the emergence of new variants and potential future coronavirus outbreaks.

In Tanzania like other African countries, health care workers (HCWs) as first responders are among the highly exposed group to SARS-CoV-2. This population group is also at high risk of exposure to any other coronaviruses infection that may be circulating within the community^15^. In this study, we investigate the heterologous nature of the neutralizing immunity against coronaviruses elicited in front line HCWs in Tanzania two years into the SARS-CoV-2 pandemic. Overall, we found different levels of neutralization capacity against the tested coronaviruses except for MERS-CoV. Additionally, we found evidence for elicitation of cross-neutralization immunity against Severe Acute Respiratory Syndrome Coronavirus 1 (SARS-CoV-1) following SARS-CoV-2 exposure.

## Methods

### Study design and participants

This was a cross-sectional study involving front line HCWs from designated COVID-19 units, emergency departments, and infectious diseases units in two tertiary hospitals in Dar es Salaam -Tanzania. Dar es Salaam is the largest and most populated urban area in Tanzania and was the heaviest hit city with COVID-19 in Tanzania according to official data^16^. This study recruited 200 exposed HCWs who were involved in treating and caring COVID-19 patients. Study participants were conveniently enrolled into the study based on their availability during the period of May and June 2022.

### Participant’s information, clinical and laboratory tests

Data on participants’ demographics, clinical information were collected using a structured questionnaire through interviews with participants’ demographic information including age, sex and departments at which they were working were collected. Clinical data such as history of symptoms (if any) relevant to COVID-19 during the month of January to June 2022, history of hospitalization, and risk information for severity of COVID-19 including cardiovascular diseases, renal and liver diseases, COPD and diabetes mellitus were collected. Laboratory test results for PCR test for SARS-CoV-2 was also collected.

### Sample collection, handling and SARS-CoV-2 serological test

Whole blood samples were collected in EDTA vacutainer tube and sent to laboratory for immediate processing within 2-h of collection. Plasma was isolated by centrifugation then immediately stored in portions at −80 °C. We used STANDARD Q COVID-19 IgM/IgG plus antibody test kit (SD Biosensor) to detect SARS-CoV-2 IgG and IgM from plasma samples of study participants. This kit is a rapid immunochromatography test designed for the qualitative presumptive detection of anti nucleocapsid (N) and antispike –RBD specific SARS-CoV-2 IgM and IgG ^17^. Briefly, 10 µL of plasma was added to the specimen well followed by 3 drops of buffer. Reading of the test results were done after 10–15 min as per manufacturer protocol. A sample was regarded as positive when the colored bands appeared on the result window corresponding to “C” Control line together with “G” and/or “M” Test line ^18^.

### SARS-CoV-2 ELISA

Anti-SARS-CoV-2 S-RBD protein Human IgG ELISA kit (Proteintech) was used for quantitative detection of anti-SARS-CoV-2 RBD IgG in plasma samples as per manufacturer’s protocol. Briefly, plasma samples and controls were diluted to 1:100, and added in duplicate to microwells pre-coated with S-RBD recombinant protein. After 30 min incubation at room temperature, the wells were washed and a horseradish peroxidase conjugated anti-human IgG were added to each well and incubated. The presence of immune complex recombinant protein-human anti-S-RBD IgG antibody-HRP conjugated antibody was demonstrated after the addition of a chromogenic solution that initiated a color development reaction. The optical density reading obtained with a spectrophotometer set at 450/620 nm was proportional to the amounts of antibodies present in the specimen. The average of the duplicate readings for each standard and sample was subtracted from the average zero standard absorbance. Anti-S-RBD antibody concentrations were obtained from the best-fit standard curve determined by regression analysis using four-parameter logistic curve fit.

### Preparation of spike-expressing plasmids

Plasmids expressing codon-optimized spike proteins of SARS-CoV (strain Frankfurt 1; GenBank accession number AY291315.1), MERS-CoV (strain EMC; GenBank accession number NC_019843.3.1), HCoV-229E (strain Inf-1; GenBank accession number NC_002645.1), HCoV-NL63 (strain Amsterdam I; GenBank accession number AY567487.2), were synthesized by a gene synthesis service (Fasmac). These plasmids were fragmented by restriction enzymes (*Kpn*I, NEB, Cat# R3142L; *Not*I, NEB, Cat# R3189L) and cloned into the *Kpn*I-*Not*I site of backbone pCAGGS vector ^19^ using T4 DNA Ligase (NEB, Cat# M0202L). To construct the plasmids expressing the receptor proteins of coronaviruses, the ORF of human alanyl aminopeptidase, membrane (ANPEP)/CD13 (GenBank accession number NM_001150.3) was prepared by RT-PCR using BjaB cell-derived cDNA as the template and the following primers: hANPEP-MluI-Fwd, 5'-tatatataACGCGTatggccaagggcttctatatttcc-3' and hANPEP-HpaI-Rev, 5'-ttaattaaGTTAACctatttgctgttttctgtgaaccactgg-3'. The ORF of human dipeptidyl peptidase-4 (DPP-4)/CD26 was subcloned from pcDNAhumanDPP4 (kindly provided by Dr. Shuetsu Fukushi) ^20^. The obtained ORF fragments were inserted into *Mlu*I-*Hpa*I site of pLV-EF1a-IRES-Puro (Addgene, Cat #85132).

### Generation of HOS-TMPRSS2 cells stably expressing a variety of human ANPEP or human DPP4 proteins

To prepare lentiviral vectors expressing human ANPEP or human DPP4, HEK293T cells (2 × 10^6^ cells) were cotransfected with 12 μg of pCAG-HIVgp, 10 μg of pCMV-VSV-G-RSV-Rev, and 17 μg of pLV-EF1a-human ANPEP-IRES-Puro or pLV-EF1a-human DPP4-IRES-Puro by the calcium phosphate method. After 12 h of transfection, the culture medium was changed to fresh medium, and then 36 h later, the culture supernatant including lentivector particles was collected. HOS-TMPRSS2 cells (100,000 cells) were then transduced with the resultant lentiviral vector. After 48 h post transduction, transduced cells were maintained for puromycin (0.5 μg/mL; Invivogen, cat# ant-pr-1) and G418 (1 mg/mL; Nacarai tesque, cat# 09380-44) selections for 14 days.

### Coronaviruses pseudovirus production

Pseudoviruses bearing SARS-CoV-2 spike protein were produced as previously described ^21^. Briefly, 293 T cells were seeded and co-transfected with 50 ng of HCoV spike-encoding plasmids and 2 µg of a previously prepared lentiviral backbone ^22,23^, carrying a luciferase reporter gene and a HiBiT peptide tag, designated as pSG3_ΔENVΔNef_-Luc2-IN/HiBit. After 48 h of incubation at 37 °C and 5% CO_2_, DNase was added to the cell supernatant to degrade any unutilized lentiviral plasmid and incubated for 30 min. The culture supernatant was clarified by spinning down at 400*g* for 5 min. Pseudovirus concentration was quantified based on HiBiT-generated luminescence and normalized by the corresponding level of p24 antigen as previously described ^22^. The same procedure was followed to produce and quantify pseudoviruses bearing NL63, 229E, SARS-CoV-1 and MERS-CoV spikes. All pseudoviruses were stored at −80 °C until needed.

### Plasma neutralization assay

Plasma was tested for SARS-CoV-2 spike neutralization as previously described^24,25^. In brief, heat inactivated plasma (56 °C for 45 min) was fivefold serially diluted on a 96-well plate from a starting dilution of 1:40. Pseudoviruses bearing SARS-CoV-2 spike were thawed and added at a concentration of 6 ng p24 antigen per well, and incubated. After 1 h incubation at 37 °C and 5% CO_2_, HOS cells stably expressing ACE2 and TMPRSS2 were added at a density of 10,000 cells/well and incubated for additional 48 h for chemiluminescence detection (ONE-Glo, Promega). Plasma neutralization was determined by calculating the percent reduction in luminescence between the test wells and pseudovirus only wells. Neutralizing titers were calculated on a dose–response curve fit with a non-linear function and expressed as NT_50_ values. The same procedure was employed to quantify neutralizing titers against other HCoVs albeit with alterations in target cells and pseudovirus concentration. Specifically, target cells for NL63 and SARS-CoV-1 spike-bearing pseudovirus infection were HOS-ACE2/TMPRSS2 cells^23,26^, and those for MERS-CoV and 229E spike-bearing pseudovirus infection were HOS-DPP4/TMPRSS2 and HOS-ANPEP /TMPRSS2 cells, respectively. Optimal pseudovirus titers for neutralization were obtained by titration on relevant target cells where 6 ng were used for pseudoviruses bearing SARS-CoV-2, SAR-CoV-1 and MERS-CoV and 8 ng for HC0V-229E and 25 ng for HCoV-NL63.

To screen for non-specific neutralization activity, pseudoviruses expressing VSV-G protein instead of coronaviruses spike were used ^27^. Samples demonstrating significant inhibition activity against VSV-G were categorized as exhibiting non-specific inhibition targeting the proviral backbone and were subsequently excluded from further analysis.

### Data management and analysis plan

The measures of central tendencies and dispersion (mean, median, range, and standard deviation) was used to summarize continuous variable from baseline characteristics of participants, antibody concentration and neutralization titers for different coronaviruses. Correlation analysis between neutralization titters was performed using the Spearman test. The Kruskal–Wallis test was used to compare medians between three or more groups, and Mann–Whitney tests were applied for comparisons between two groups. All Data and statistical analysis were performed in GraphPad Prism version 7 and a p-value of < 0.05 was considered significant.

### Ethics approval

Ethical approval for this study was obtained from the Muhimbili University of Health and Allied Sciences Senate Research and Publications Committee (MUHAS-REC-12-2020-437), the National Institute for Medical Research (NIMR) (NMR/HQ/R.8a/Vol/IX/3789) in Tanzania and Kumamoto University (#2170), Japan. Written informed consent was obtained from each study participant. All methods applied in this study complied with the Declaration of Helsinki for medical research involving human subjects.

## Results

### Clinical and social demographic characteristics of the participants

A total of 200 healthcare workers (HCWs) were recruited from Muhimbili National Hospital and Aga Khan Hospital, where the majority (64.5%) were female, and only 16% were above the age of 40 years. All study participants were considered to be highly exposed to SARS-CoV-2 in the past 6 months prior to recruitment, given the aforementioned hospitals were designated to handle COVID-19 cases in the wider Dar es Salaam metropolis. Only 39 (19.5%) of the HCWs had been COVID-19 vaccinated with the majority, 33 (84.6%), having received the primary series (single dose) of the Ad26.COV2.S (Table 1).

**Table 1:** Clinical and demographic characteristics of recruited HCW.

Characteristics	N	%
Sex		
Male	71	35.5
Female	129	64.5
Hospital		
Private	102	51
Public	98	49
Age group		
18–29	111	55.5
30–39	57	28.5
40–49	26	13
> 50	6	3
Department		
EMD	90	45
ICU	49	24.5
INTM	49	24.5
OPD	1	0.5
PHYSIO	11	5.5
Carder		
Doctor	36	18
Nurse	68	34
Other	5	2.5
Support	91	45.5
Comorbidity		
Yes	18	9
No	182	91
Exposure to SARS CoV-2		
Yes	200	100
No	0	0
Anti SARS COV-2 IgG		
Positive	188	94
Negative	12	6
Vaccination status		
Yes	39	19.5
No	171	85.5
Vaccine type (n = 39)		
Ad26.COV2.S	33	84.6
Ad26.COV2.S / BNT162b2^a^	1	2.6
mRNA-1273^a^	1	2.6
BNT162b2^a^	3	7.7
Sputnik	1	2.6

*EMD* emergency department, *ICU* intensive care unit, *INTM* internal medicine, *OPD* outpatient department, *PHYSIO* physiotherapy department.

^a^Original monovalent vaccines.

Seropositivity for SARS-CoV-2 IgG and IgM was found in 188 (94%) and 2 (1%) participants, respectively, suggesting past (and current) infection in most of the HCWs (Table 1). These results were further confirmed by ELISA detection of anti-RBD binding antibodies in 196 (98%) participants. Based on COVID-19 like symptoms experienced in the last 6 months of sample collection, 0 (0%) HCW reported serious illness requiring critical care, 6 (3%) reported COVID-19-like illness that required hospitalization, while 43 (21.5%) reported mild symptoms that required over the counter medication.

### Neutralising activity against coronaviruses in HCWs samples

**Figure 1:**
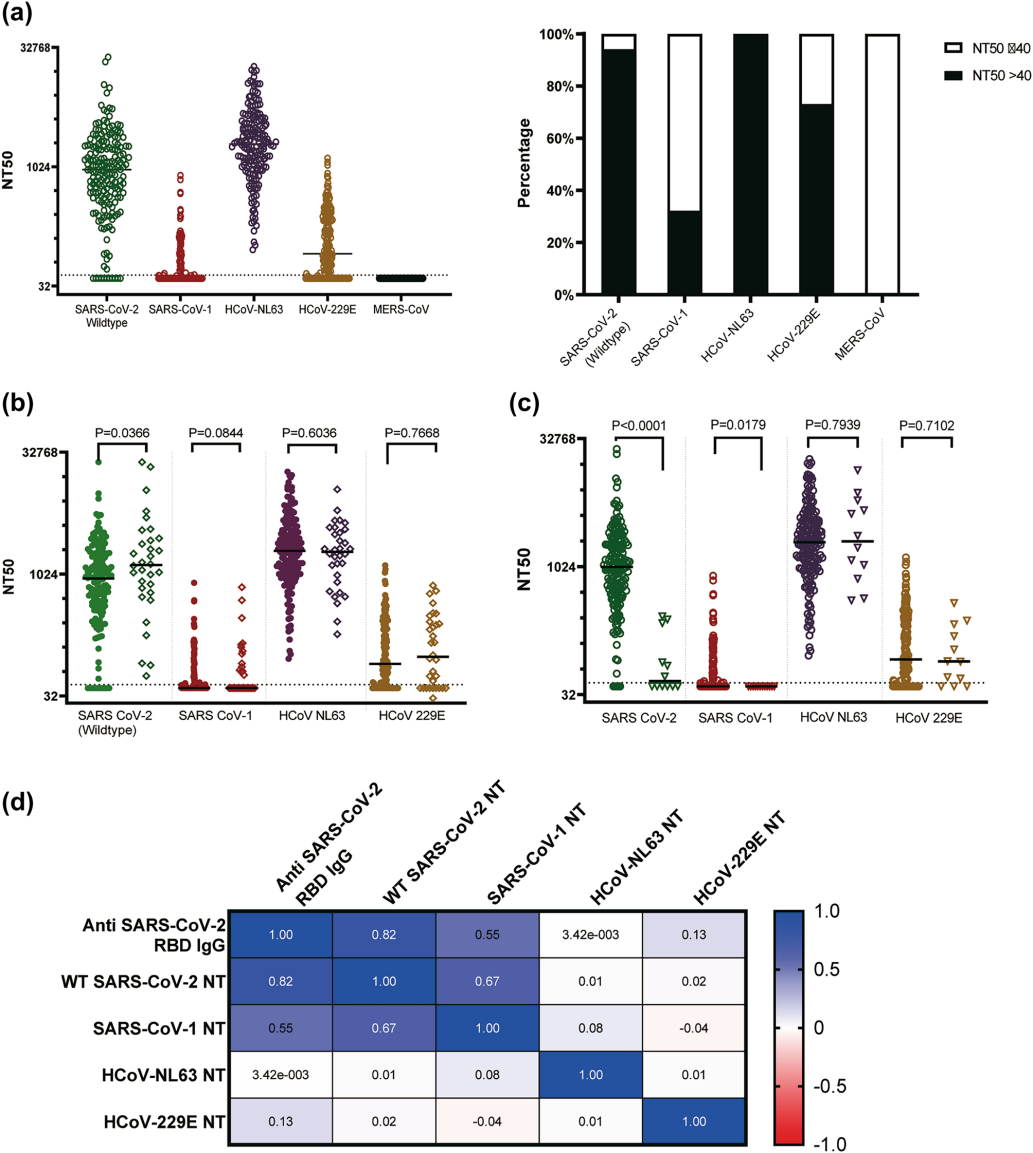
Neutralization capacity of plasma (n = 186) form HCWs against coronaviruses spikes pseudotyped viruses. (**a**) Neutralisation profile of plasma against SARS-CoV-2 wildtype, SARS-CoV-1, MERS-CoV, HCoV-229E, and HCoV-NL63. (**b**) Comparison of neutralization titers between SARS-CoV-2 exposed-vaccinated (indicated by solid circles symbols) and exposed-unvaccinated (open diamond symbols), and (**c**) between SARS-CoV-2 seropositive (circle symbols) and seronegative groups (triangle symbols). The horizontal dotted line indicates the lowest limit of dilution tested (1:40). Statistical significance was determined using the Mann–Whitney-U rank test and P value < 0.05 was considered significant, (**d**) Spearman's correlation between neutralization titers for coronaviruses and the total anti-SARS-CoV-2 RBD binding antibodies quantified by ELISA. *WT* wild-type, *NT* neutralization titer.

To quantify the neutralizing capacity of antisera from HCWs, we measured the dilution of antisera required to prevent 50% infectivity of pseudoviruses expressing spike proteins for SARS-CoV-2, SARS-CoV-1, MERS-CoV, and HCoV NL63 and 229E. Samples with neutralization titer (NT_50_) of over 1 in 40 dilution were classed as neutralizing. For this analysis 14 sera samples showed non-specific inhibition activity and were excluded for the analysis. Of the remaining 186 plasma, nearly all samples (94%) showed a potent neutralization activity against SARS-CoV-2 wild-type (Wuhan ancestral) spike with median NT_50_ of 942. Similarly, a robust neutralising activity was observed in 100% of the samples against NL63 spike with median NT_50_ of 1980. On the other hand, we observed lower neutralisation activity against 229E spike in 67% of sera samples with median NT_50_ of 82. Only 32.2% of HCWs sera could neutralise SARS-CoV-1 spike at NT_50_ ranging from 41 to780 while none of the samples showed neutralising activity against MERS-CoV spike (Fig. 1a).

We next assessed whether vaccination status could impact SARS-CoV-2 neutralization in these highly exposed HCWs. We found that median neutralisation activity was significantly higher against SARS-CoV-2 wild-type spike (p = 0.0366), while it was modestly high, but not statistically significant, against SARS-CoV-1 (p = 0.0844) in the exposed vaccinated HCW sera. In contrast, there were no differences in neutralisation capacity between exposed vaccinated and exposed unvaccinated sera against the tested seasonal coronaviruses (Fig. 1b).

If infection with SARS-CoV-2 increased the neutralising antibody titer for other coronaviruses, we would expect a higher neutralization in SARS-COV-2 seropositive samples. When we compared neutralisation between serostatus groups, we found that seropositive group showed higher neutralising activity against SARS-CoV-1 while seronegative group showed no neutralisation activity (p = 0.0179). In contrast, both human coronaviruses NL43 and 229E showed no differences in neutralisation between SARS-CoV-2 seropositive and negative groups (Fig. 1c). We found a strong correlation between neutralisation of SARS-CoV-2 and SARS-CoV-1 suggesting cross-neutralizing immunity between the two coronaviruses. No correlation was observed between human seasonal coronaviruses and SARS-CoV-2 or SARS-CoV-1 (Fig. 1d).

### Cross neutralising capacity against Omicron subvariants

**Figure 2:**
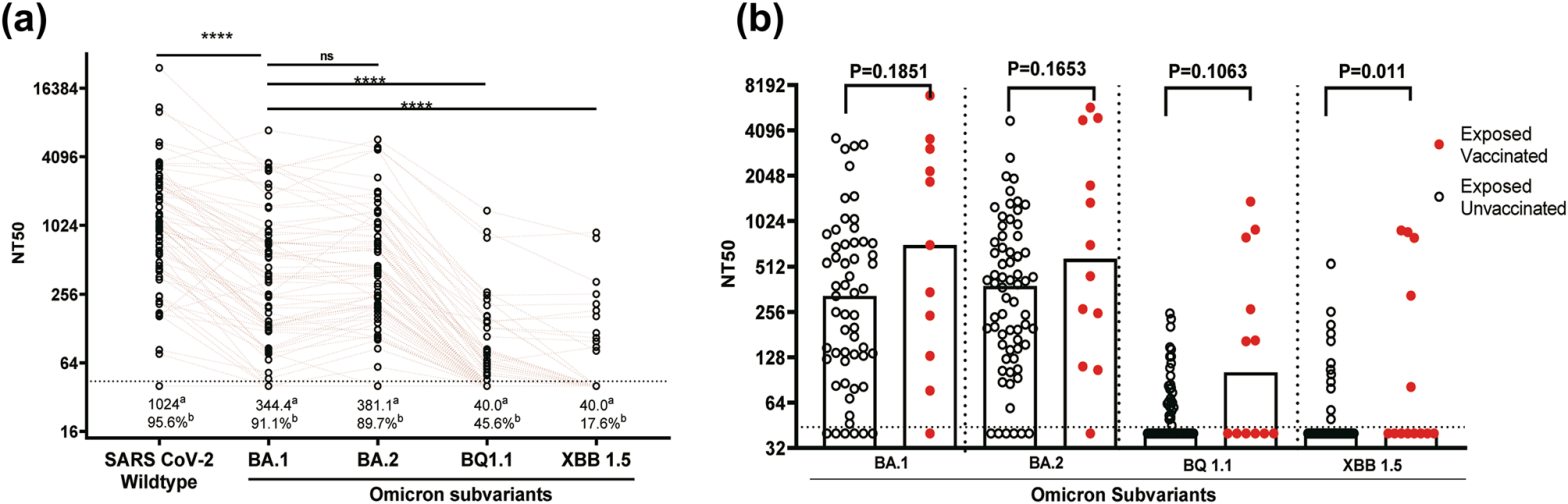
Cross-neutralization of HCWs' plasma against newer omicron subvariants. (**a**) Matched paired analysis comparing neutralization titers for HCWs' plasma (n = 68) against wild-type SARS-CoV-2 and Omicron subvariants. “a” median NT50, “b” percentage of samples with NT50 > 40. Statistical significance was determined using the Wilcoxon test (****: P < 0.0001). (**b**) Comparison of neutralization titers across Omicron subvariants between SARS-CoV-2-exposed-vaccinated and SARS-CoV-2-exposed-unvaccinated HCWs. Statistical significance was determined using the Mann–Whitney-U rank test. In (a**,b**), the horizontal dotted line indicates the lowest limit of dilution tested (1:40).

During the data collection for this study, Tanzania and neighbouring countries were experiencing the end of the first wave of Omicron variant ^16,28^. Therefore, we aimed to determine the cross-neutralizing immunity against the newer Omicron subvariants, i.e., BQ.1.1 and XBB.1.5, which were spreading in other parts of the world. A total of 68 plasma samples were analysed for neutralisation against the selected Omicron subvariants’ spikes. Compared to the wild-type, Omicron subvariants showed increasing resistance to neutralization (p < 0.0001) (Fig. 2a). When compared to BA.1 and BA.2, the plasma from HCWs showed a significant decrease in neutralization activity against BQ.1.1 and XBB.1.5.

To assess whether vaccination status had impact on neutralizing immunity against the tested Omicron subvariants, the serum samples were grouped into two categories: exposed vaccinated and exposed unvaccinated groups. Across the subvariants, exposed vaccinated group showed higher neutralization titers compared to exposed unvaccinated groups. This trend was statistically significant in XBB.1.5 subvariants (Fig. 2b), suggesting a tendency towards enhanced neutralizing immunity in HCWs who were exposed and received at least one dose of the COVID-19 vaccine.

## Discussion

The recent SARS-CoV-2 pandemic and previous outbreaks highlight the health threat posed by coronaviruses. While the WHO has declared the end of the COVID-19 emergency, our understanding on how the current pandemic and previous exposures to endemic and zoonotic coronaviruses have shaped our immunity against this family of viruses remain to be studied. In our study, we investigated the heterologous nature of neutralizing antibodies against coronaviruses in Tanzanian HCWs who during the height of the pandemic, represented one of the most exposed groups to SARS-CoV-2 infection ^15^. We found that nearly all HCWs had developed a robust neutralizing immunity against SARS-CoV-2 spike, with indications of cross-neutralization activity against SARS-CoV-1 but not against the tested HCoVs. Similar to other studies ^29–31^, hybrid immunity in this highly SARS-CoV-2 exposed group was associated with enhanced cross-neutralization activity of the elicited neutralizing immunity.

Overall the HCWs' sera demonstrated potent pseudovirus neutralization activity against the wild-type SARS-CoV-2 spike. This activity was strongly correlated with RBD binding antibody titers. Exposure to human and emerging coronaviruses may diversify adaptive immunity against SARS-CoV-2 ^1,32–35^. In this study, our analysis of the relationship between neutralization activity against SARS-CoV-2 and that of coronaviruses (SARS-CoV-1 and HCoV-NL63)^36^, which share ACE2 as their cellular receptor, showed a strong association only with SARS-CoV-1 neutralizing activity. Although all sera showed potent neutralization activity against HCoV-NL63, we did not find any association between this activity and that of SARS-CoV-2, suggesting a lack of elicitation of heterologous neutralizing activity against SARS-CoV-2 from exposure to HCoV-NL63. Additionally, over two-thirds of sera samples in our study showed neutralizing activity against another alpha coronavirus, HCoV-229E, but this activity was not correlated with that against SARS-CoV-2. These findings are in line with the observations that exposure to human alphacoronaviruses is not associated with elicitation of cross-neutralizing immunity against SARS-CoV-2^37,38^.

On the flip side, one of our goal was to clarify whether the ongoing exposure to SARS-CoV-2 could boost immune responses to previously encountered coronaviruses or induce heterologous immunity against other coronaviruses. We demonstrate that there was a strong correlation between SARS-CoV-2 RBD binding antibody titers and neutralization activity against SARS-CoV-1 which is expected, given the substantial relatedness and sequence similarity, particularly within the RBD of both viruses^39^. Additionally, seronegative samples for SARS-CoV-2 showed no neutralization activity against SARS-CoV-1, suggesting that the observed neutralizing activity could be secondary to cross-neutralization activity from SARS-CoV-2-induced antibodies. However, pre-pandemic samples from African cohorts have shown the ability, albeit mild, to neutralize SARS-CoV-1 spike^2^, thus the observed activity against SARS-CoV-1 may not be entirely explained by cross-neutralization by SARS-CoV-2-induced antibodies. Nonetheless, our data demonstrate the potential for heterologous neutralizing immunity against SARS-CoV-1 that could be elicited following coronavirus infections. In a study by Alkhalifah et al., they demonstrated the boosting effect of IgG levels against MERS-CoV following exposure to SARS-CoV-2 infection^40^. On the other hand, neutralizing activity against MERS-CoV-2 pseudoviruses was demonstrated in SARS-CoV-2 convalescent sera from one Chinese study^41^. In our study, none of the sera showed any neutralization activity against this coronavirus at a dilution of 1:40, suggesting a lack of exposure or elicitation of heterologous immunity to this virus in our study population. The absence of differences in neutralization activity against NL63 and 229E between SARS-CoV-2 serostatus groups, as observed in our study, could suggest the lack of reactivation of memory HCoVs B cells, contrary to that reported in other studies^42^. However, a specially designed study is needed to clarify this observation.

The constantly emerging SARS-CoV-2 variants have demonstrated a significant ability to evade humoral immunity elicited by vaccination or previous infection with older variants^43,44^. Our study revealed that HCWs sera had a significantly reduced neutralization capacity against the newer Omicron subvariants XBB1.5 and BQ1.1. However, our data suggest that a higher neutralization capacity against the wildtype SARS-CoV-2 is also associated with higher neutralization activity against emerging variants. Since the vaccination status in our study population was associated with higher neutralization titers, our results suggest that even in these highly SARS-CoV-2-exposed HCWs, there may be some benefits from vaccination with generic vaccine in improving the level of protective antibody titers against newer variants.

Our study has some limitations. First, it was a cross-sectional study involving HCWs in Tanzania, making it impossible to determine the timing of infection and the specific SARS-CoV-2 variants involved. Our panel of coronavirus spikes, tested for their neutralization, did not include all human coronaviruses, consequently, the extent of heterologous humoral immunity elicited could not be fully elucidated. Moreover, our study solely focused on neutralizing immunity against coronaviruses, and therefore, we cannot exclude the potential presence of heterologous T-cell immunity that might have been elicited following exposure to different coronaviruses. Nevertheless, our study contributes to the documentation of neutralizing immunity against coronaviruses in an understudied population whose potential exposure to other coronaviruses remains largely undescribed.

## Data Availability

The data set obtained in this study are available upon request to the corresponding author.
